# Triplications of human chromosome 21 orthologous regions in mice result in expansion of megakaryocyte-erythroid progenitors and reduction of granulocyte-macrophage progenitors

**DOI:** 10.18632/oncotarget.23463

**Published:** 2017-12-19

**Authors:** Chunhong Liu, Tao Yu, Zhuo Xing, Xiaoling Jiang, Yichen Li, Annie Pao, Justin Mu, Paul K. Wallace, George Stoica, Andrei V. Bakin, Y. Eugene Yu

**Affiliations:** ^1^ The Children's Guild Foundation Down Syndrome Research Program, Genetics and Genomics Program and Department of Cancer Genetics and Genomics, Roswell Park Cancer Institute, Buffalo, NY 14263, USA; ^2^ Department of Medical Genetics, Tongji Medical College, Huazhong University of Science and Technology, Wuhan, Hubei 430030, China; ^3^ Department of Flow and Image Cytometry, Roswell Park Cancer Institute, Buffalo, NY 14263, USA; ^4^ Department of Pathobiology, Texas A&M University, College Station, TX 77843, USA; ^5^ Genetics and Genomics Program and Department of Cancer Genetics and Genomics, Roswell Park Cancer Institute, Buffalo, NY 14263, USA; ^6^ Genetics, Genomics and Bioinformatics Program, State University of New York at Buffalo, Buffalo, NY 14263, USA

**Keywords:** Down syndrome, myeloproliferative disorder, genetically engineered mouse models, megakaryocyte-erythrocyte progenitors, granulocyte-monocyte progenitors

## Abstract

Individuals with Down syndrome (DS) frequently have hematopoietic abnormalities, including transient myeloproliferative disorder and acute megakaryoblastic leukemia which are often accompanied by acquired GATA1 mutations that produce a truncated protein, GATA1s. The mouse has been used for modeling DS based on the syntenic conservation between human chromosome 21 (Hsa21) and three regions in the mouse genome located on mouse chromosome 10 (Mmu10), Mmu16 and Mmu17. To assess the impact of the dosage increase of Hsa21 gene orthologs on the hematopoietic system, we characterized the related phenotype in the *Dp(10)1Yey/+;Dp(16)1Yey/+;Dp(17)1Yey/+* model which carries duplications spanning the entire Hsa21 orthologous regions on Mmu10, Mmu16 and Mmu17, and the *Dp(10)1Yey*/+;*Dp(16)1Yey*/+;*Dp(17)1Yey/+*;*Gata1^Yeym2^* model which carries a *Gata1s* mutation we engineered. Both models exhibited anemia, macrocytosis, and myeloproliferative disorder. Similar to human DS, the megakaryocyte-erythrocyte progenitors (MEPs) and granulocyte-monocyte progenitors (GMPs) were significantly increased and reduced, respectively, in both models. The subsequent identification of all the aforementioned phenotypes in the *Dp(16)1Yey/*+ model suggests that the causative dosage sensitive gene(s) are in the Hsa21 orthologous region on Mmu16. Therefore, we reveal here for the first time that the human trisomy 21-associated major segmental chromosomal alterations in mice can lead to expanded MEP and reduced GMP populations, mimicking the dynamics of these myeloid progenitors in DS. These models will provide the critical systems for unraveling the molecular and cellular mechanism of DS-associated myeloproliferative disorder, and particularly for determining how human trisomy 21 leads to expansion of MEPs as well as how such an alteration leads to myeloproliferative disorder.

## INTRODUCTION

Human trisomy 21, the most common human aneuploidy compatible with postnatal survival, occurs in approximately one out of 700–1000 live births [1, 2]. This chromosomal anomaly causes a constellation of developmental abnormalities, classified as Down syndrome (DS). The major clinical manifestations of DS include cardiovascular malformations, craniofacial abnormalities, developmental cognitive deficits, hematopoietic abnormalities, and Alzheimer-type neurodegeneration with variable penetrance and onset [3, 4].

Among hematopoietic abnormalities, transient myeloproliferative disorder (TMD) occurs in a substantial percentage of children with DS, and many of these patients later develop acute megakaryoblastic leukemia (AMKL) [5]. It has been demonstrated that children with DS who develop TMD and AMKL have acquired somatic mutations in exon 2 of the *GATA1* gene on the X chromosome, which lead to the generation of a mutant protein, GATA1s [6, 7]. The *GATA1s* germline mutation is associated with impaired erythropoiesis [8, 9].

**Figure 1:**
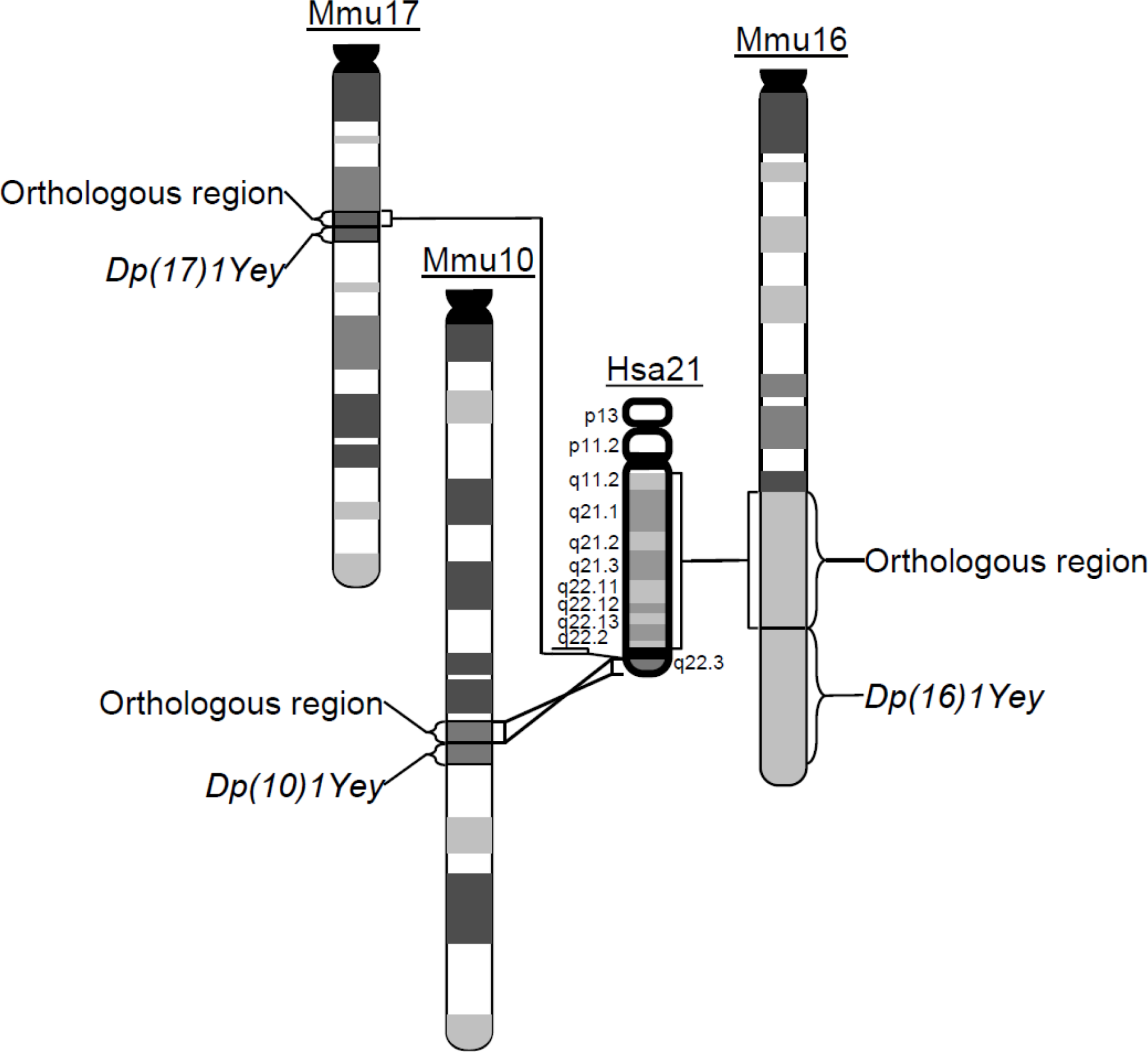
The schematic representation of the chromosomal alterations in *Dp(10)1;Dp(16)1;Dp(17)1* mice Generated using Cre/*loxP*-mediated chromosome engineering, *Dp(10)1;Dp(16)1;Dp(17)1* mice contain three duplications spanning the entire Hsa21 orthologous regions on Mmu10, Mmu16 and Mmu17, respectively.

To unravel the mechanism underlying development of myeloproliferative disorder in DS, genetic models with phenotypes mimicking DS at the molecular, cellular and organ levels are necessary. The mouse has been the most important model organism for DS due to evolutionary conservation between the human and mouse genomes. Regions on human chromosome 21 (Hsa21) are syntenically conserved with regions on mouse chromosome 10 (Mmu10), Mmu16 and Mmu17, which contain 41, 115, and 19 Hsa21 gene orthologs, respectively (Figure 1). DS is a contiguous gene syndrome [10] and evidence indicates that, for many DS phenotypes, more than one triplicated gene contributes through direct actions and/or interactions of the triplicated genes [11–17]. Therefore, in a mouse model, triplication of more Hsa21 gene orthologs increases the probability that all the direct actions and/or interactions that have a significant effect on a specific DS phenotype are mimicked.

Myeloproliferative disorder has been observed in several mouse models of DS [18–21]. Among them, Ts65Dn is the first viable trisomic mouse model of DS. This mutant carries Ts(17^16^)65Dn, an unbalanced derivative of a balanced chromosomal translocation, which was randomly induced by irradiation at Muriel Davisson's laboratory [22, 23]. Tc1 is another important mouse model of DS, developed by introducing Hsa21 into mouse embryonic stem (ES) cells using microcell-mediated chromosome transfer [24–27]. The hematopoietic phenotype has been extensively characterized in the Ts65Dn and Tc1 mouse models [18, 19]. However, a substantial number of Hsa21 gene orthologs are not triplicated in either model. To include those missed Hsa21 orthologs, we have generated *Dp(10)1Yey/+* [i.e. *Dp(10)1*]*, Dp(16)1Yey/+* [i.e. *Dp(16)1*], and *Dp(17)1Yey/+* [i.e. *Dp(17)1*] mice, by chromosome engineering which carry duplications spanning the entire Hsa21 orthologous regions on Mmu10, Mmu16, and Mmu17, respectively; thus all the DS-associated gene dosage alterations are mimicked (Figure 1) [15, 28]. We have shown that *Dp(10)1;Dp(16)1;Dp(17)1* and *Dp(16)1* mice exhibited DS-related heart defects, cognitive behavioral deficits, and impaired hippocampal long-term potentiation [15]. Extensive studies are also being performed on these mutant mice by many other laboratories [10, 29–38]. In this study, we extended the exploration of the impact of these engineered gene dosage alterations to DS-related hematopoiesis.

## RESULTS

### Generation of *Gata1^Yeym2^* mice to delete exon 2 of the *Gata1* gene

**Figure 2:**
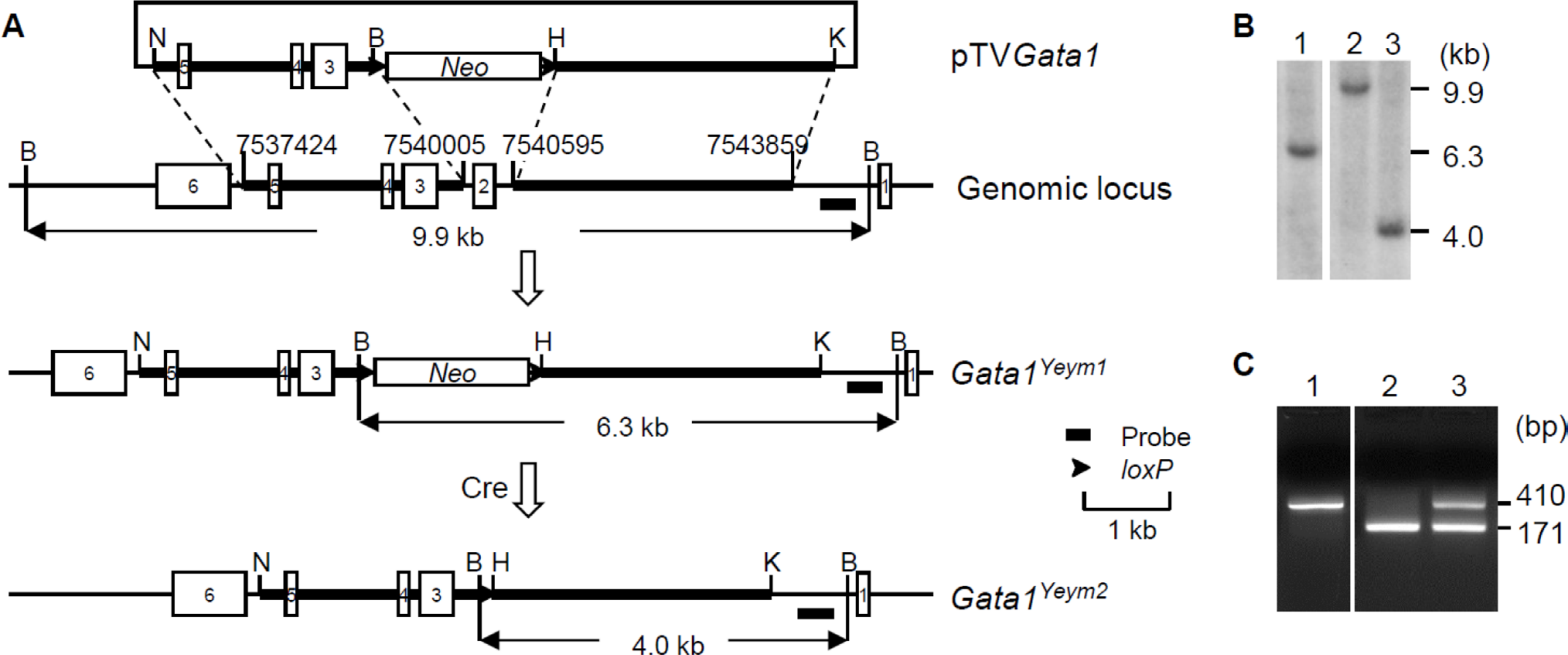
Development of *Gata1^Yeym2^* mutant mouse strain (**A**) Targeting strategy for generating the *Gata1^Yeym2^* allele. *Neo*, neomycin-resistance gene cassette. B, *Bam*HI; H, *Hind*III; K, *Kpn*I; N, *Not*I. The genome coordinates of the homology arms were marked. (**B**) Southern blot analysis of *Bam*HI-digested ES cell DNA. Lane 1, An ES cell clone containing the *Gata1^Yeym1^* allele; lane 2, the wild-type ES cells; lane 3, the ES cell clone containing the *Gata1^Yeym2^* allele. (**C**) RT-PCR analysis of total RNA isolated from the fetal livers of E14.5 embryos, showing the PCR products from the cDNA of wild type *Gata1* and *Gata1^Yeym2^*. MW, molecular-weight size marker, TrackIt™ 50 bp DNA Ladder (Invitrogen); lane 1, a wild-type embryo; lane 2, a male *Gata1^Yeym2^* embryo; lane 3, a heterozygous female *Gata1^Yeym2^* embryo.

To assess the effect of GATA1s in DS-associated hematopoietic abnormalities, we used gene targeting to delete exon 2 of the *Gata1* gene in mouse ES cells. First, we generated ES cell lines that carried the *Gata1^Yeym1^* mutant allele using the replacement vector pTV*Gata1.* Recombination between pTV*Gata1* and the *Gata1* locus in ES cells led to the replacement of a genomic region containing exon 2 of the gene with a neomycin-resistance gene cassette flanked by two *loxP* sites (Figure 2A). We then expressed the Cre recombinase transiently in two *Gata1^Yeym1^* ES cell clones and the neomycin-resistance gene cassette was removed by Cre/*loxP*-mediated recombination. The excised allele was designated as *Gata1^Yeym2^* (Figure 2A). Germline transmission was established for four ES cell lines that carried *Gata1^Yeym2^*. Southern blot analysis was performed to confirm the deletion of the exon 2 region (Figure 2B). Expression of the mutant allele in the *Gata1^Yeym2^* mice was confirmed by RT-PCR (Figure 2C).

### Triplications of all Hsa21 orthologous regions in mice result in peripheral blood abnormalities

*Dp(10)1;Dp(16)1;Dp(17)1* and *Dp(10)1;Dp(16)1;Dp(17)1;Gata1^Yeym2^* mice were generated as described in Materials and Methods. Peripheral complete blood counts (CBCs) of *Dp(10)1;Dp(16)1;Dp(17)1* mice, *Dp(10)1;Dp (16)1;Dp(17)1;Gata1^Yeym2^* mice and their wild-type controls were measured every 3 months until 15 months of age.

**Figure 3:**
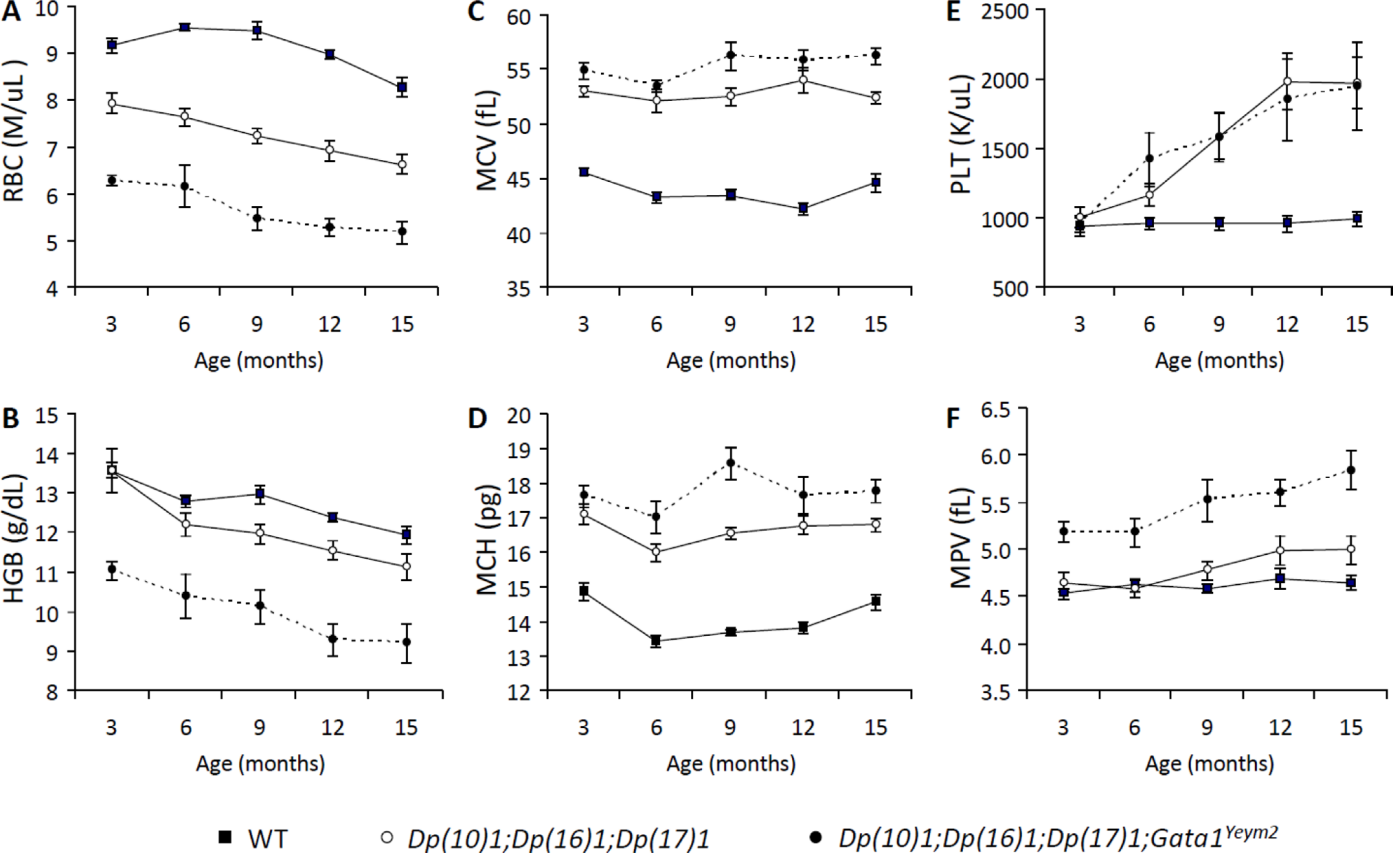
Complete blood counts (CBC) of *Dp(10)1;Dp(16)1;Dp(17)1*, *Dp(10)1;Dp(16)1;Dp(17)1;Gata1^Yeym2^* mice and wild-type controls (**A**) Red blood cells (RBC) (M/μL; millions per microliter); (**B**) Hemoglobin (HGB) (g/dL, grams per deciliter); (**C**) Mean corpuscular volume (MCV) (fL, fentoliters); (**D**) Mean corpuscular hemoglobin (MCH) (pg, picograms); (**E**) Platelets (PLT) (K/μL; thousands per microliter); (**F**) Mean platelet volume (MPV). Closed square, the wild-type controls (*n* = 14, 14, 9, 15, 21 at 3, 6, 9, 12, 15 months of age, respectively); open circle, *Dp(10)1;Dp(16)1;Dp(17)1* mice (*n* = 6, 13, 9, 14, 14 at 3, 6, 9, 12, 15 months of age, respectively); closed circle, *Dp(10)1;Dp(16)1;Dp(17)1;Gata1^Yeym2^* mice (*n* = 8, 5, 5, 6, 5 at 3, 6, 9, 12, 15 months of age, respectively).

CBCs revealed reduced counts of red blood cells (RBCs) starting at 3 months of age (*p* < 0.001) and lowered hemoglobin concentrations (HGB) starting at 9 months of age (*p* < 0.05) in both *Dp(10)1;Dp(16)1;Dp(17)1* mice and *Dp(10)1;Dp(16)1;Dp(17)1;Gata1^Yeym2^* mice (Figure 3A–3B), which indicated that both mutants developed anemia. *Dp(10)1;Dp(16)1;Dp(17)1;Gata1^Yeym2^* mice showed much lower RBC counts (*p* < 0.01) and HGB concentrations (*p* < 0.01) than *Dp(10)1;Dp(16)1;Dp(17)1* mice (Figure 3A–3B), suggesting that the *Gata1s* mutation causes more severe anemia by further reducing the RBC counts and HGB concentrations.

CBCs also showed increased mean corpuscular volume (MCV) (*p* < 0.0001) and mean corpuscular hemoglobin (MCH) (*p* < 0.0001) in both *Dp(10)1;Dp(16)1;Dp(17)1* mice and *Dp(10)1;Dp(16)1;Dp(17)1;Gata1^Yeym2^* mice starting at 3 months of age (Figure 3C–3D), indicating that both mutants developed macrocytosis. However, MCV and MCH were similar in *Dp(10)1;Dp(16)1;Dp(17)1* mice and *Dp(10)1;Dp(16)1;Dp(17)1*;*Gata1^Yeym2^* mice (Figure 3C–3D), indicating that the *Gata1s* mutation did not contribute to increases in the volume of red blood cells.

In addition, platelet counts were elevated in both *Dp(10)1;Dp(16)1;Dp(17)1* mice and *Dp(10)1;Dp(16)1;Dp(17)1;Gata1^Yeym2^* mice starting at 6 months of age (*p* < 0.05), indicating thrombocytosis. There was no significant difference in the magnitude of the thrombocytosis between the mutants (Figure 3E), suggesting that the *Gata1s* mutation did not significantly alter the severity of this phenotype.

Mean platelet volume was significantly increased in *Dp(10)1;Dp(16)1;Dp(17)1;Gata1^Yeym2^* mice starting at 3 months of age (*p* < 0.001), but not in *Dp(10)1; Dp(16)1;Dp(17)1* mice (Figure 3F). This result suggests that the *Gata1s* mutation, but not the triplications of the Hsa21 orthologous regions, underlies the expanded platelet volume.

### Triplications of all Hsa21 orthologous regions in mice result in abnormalities in spleen and bone marrow

**Figure 4:**
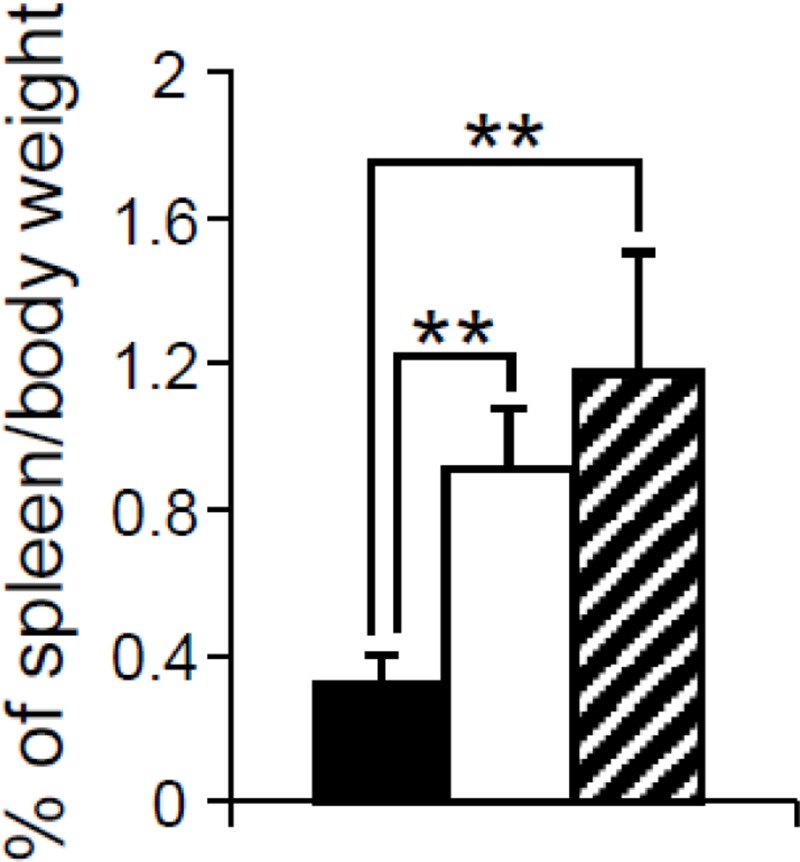
Relative weights of the spleens from mice with different genotypes The percentage of spleen weight over body weight in the wild-type controls (*n* = 6, solid bar); *Dp(10)1;Dp(16)1;Dp(17)1* mice (*n* = 6, open bar) and *Dp(10)1;Dp(16)1;Dp(17)1;Gata1^Yeym2^* mice (*n* = 5, diagonal-lined bar). ^**^p < 0.01.

To investigate the hematopoietic features of *Dp(10)1;Dp(16)1;Dp(17)1* and *Dp(10)1;Dp(16)1;Dp(17)1;Gata1^Yeym2^* mice, 15 to 24 month-old mutant mice and the wild-type littermates were euthanized and their spleens and bone marrows were harvested for histology and flow cytometry analysis. The average weights of the spleens, after normalization to body weight, accounted for 0.91 ± 0.16%, 1.18 ± 0.32% and 0.32 ± 0.08% of the total body weights in *Dp(10)1;Dp(16)1;Dp(17)1*, *Dp(10)1;Dp(16)1;Dp(17)1;Gata1^Yeym2^* and the wild-type control mice, respectively (Figure 4). These data indicate that both mutants exhibit splenomegaly (*p* < 0.01) but there is no significant difference between the weights of the spleens isolated from the two mutants (*p* > 0.05).

**Figure 5:**
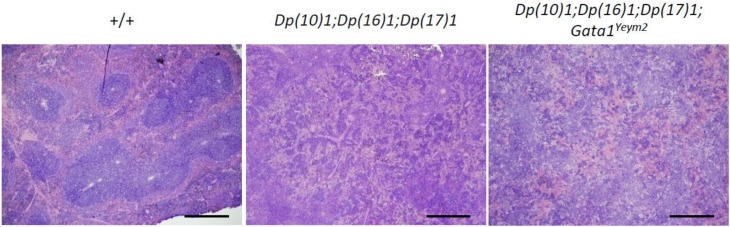
Histological sections of the spleens from different genotypes at lower magnification The spleen structure of wild-type controls, *Dp(10)1;Dp(16)1;Dp(17)1* mice and *Dp(10)1;Dp(16)1;Dp(17)1;Gata1^Yeym2^* mice under hematoxylin and eosin staining. Scale bar, 40 μm.

**Figure 6:**
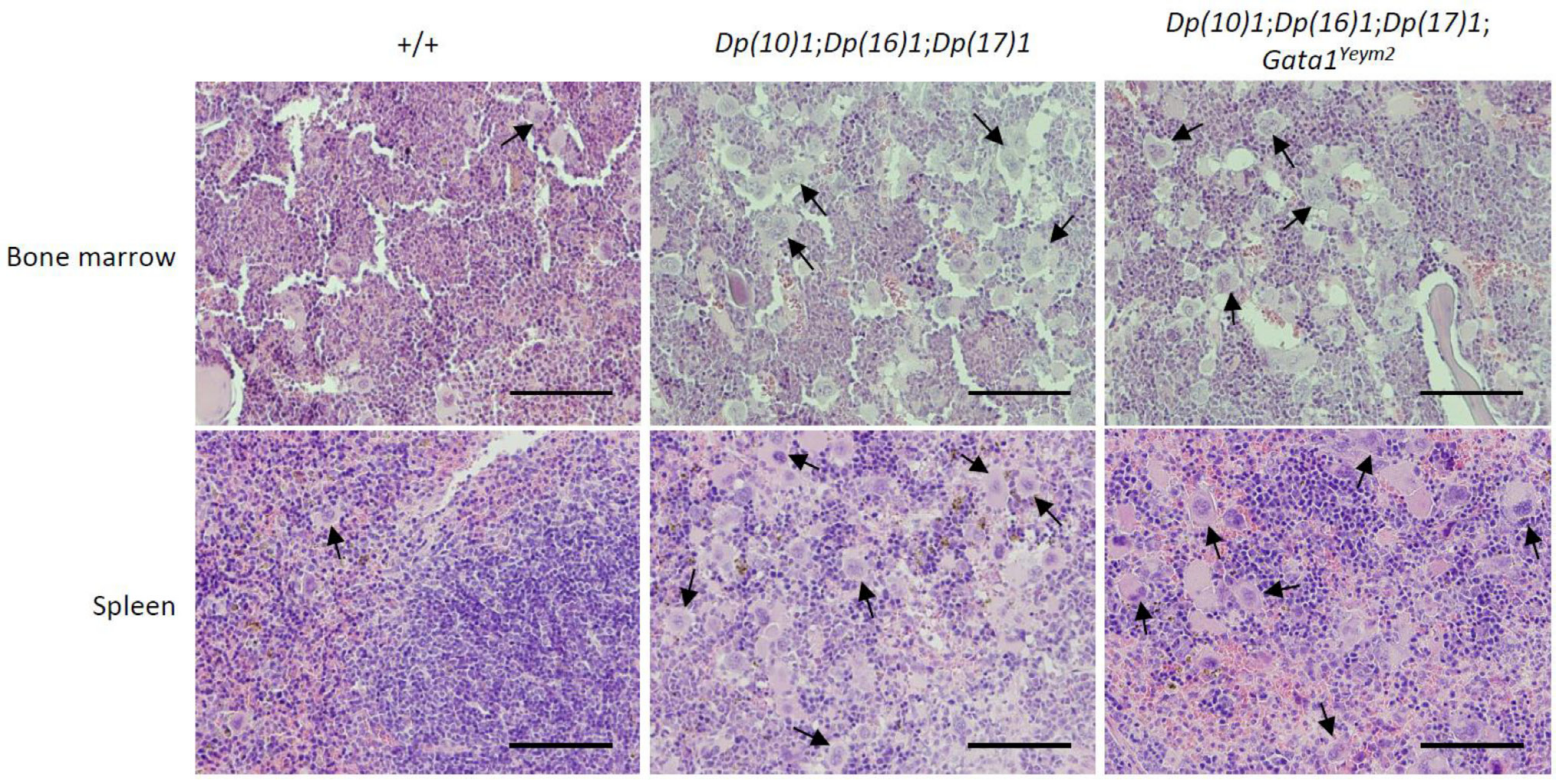
Histological sections of the bone marrow and spleens from different genotypes at higher magnification Hematoxylin and eosin staining of bone marrow and spleen sections in wild-type controls, *Dp(10)1;Dp(16)1;Dp(17)1* mice and *Dp(10)1;Dp(16)1;Dp(17)1;Gata1^Yeym2^* mice. Scale bar, 10 μm. Arrows point to the megakaryocytes in the sections.

Histological sections showed disruption of red and white pulp architecture in the spleens from *Dp(10)1;Dp(16)1;Dp(17)1* mice and *Dp(10)1;Dp(16) 1;Dp(17)1;Gata1^Yeym2^* mice (Figure 5). The structural distortions of the spleens are apparently due to severe megakaryocyte infiltration (Figure 6). The bone marrows of both *Dp(10)1;Dp(16)1;Dp(17)1* mice and *Dp(10)1;Dp(16)1;Dp(17)1;Gata1^Yeym2^* mice were also infiltrated with large numbers of megakaryocytes (Figure 6).

**Figure 7:**
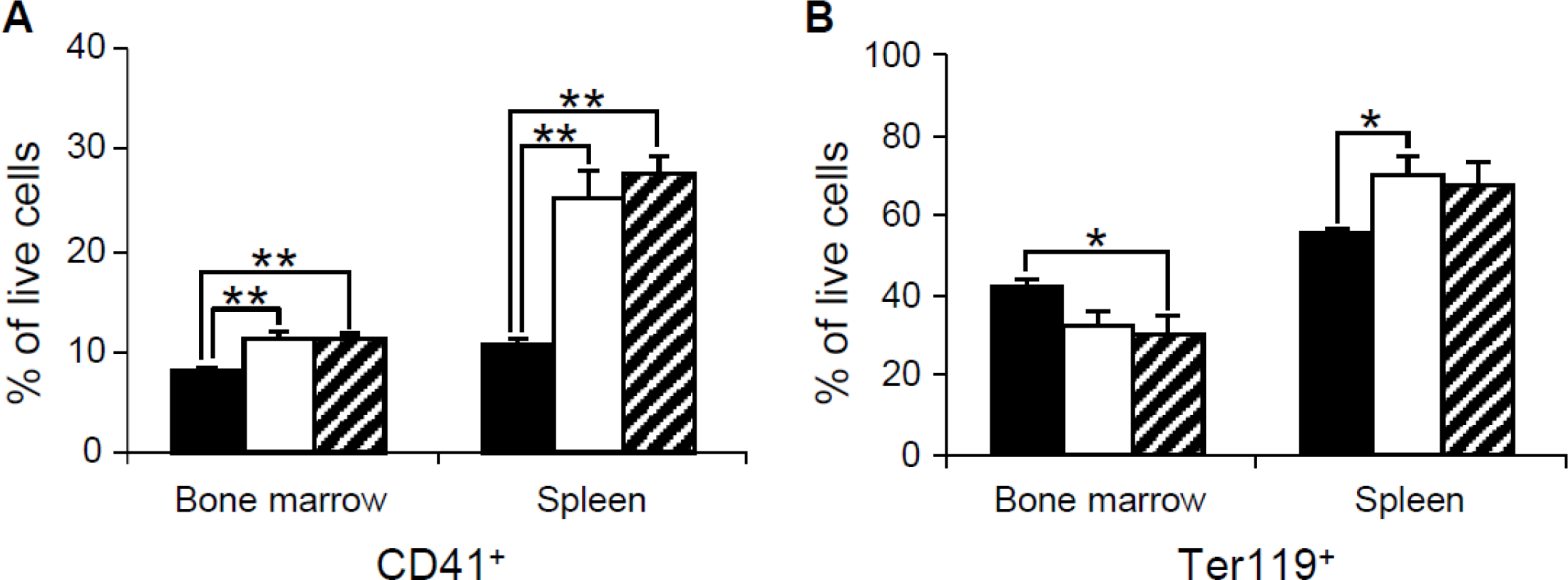
Flow cytometric analysis of bone marrow and spleen cells in wild-type controls, *Dp(10)1;Dp(16)1;Dp(17)1* mice and *Dp(10)1;Dp(16)1;Dp(17)1*;*Gata1^Yeym2^* mice (**A**) CD41^+^ megakaryocyte lineage cells; (**B**) Ter119^+^ erythrocyte lineage cells. Solid bar, the wild-type controls (*n* = 6); open bar, *Dp(10)1;Dp(16)1;Dp(17)1* mice (*n* = 6) and diagonal-lined bar, *Dp(10)1;Dp(16)1; Dp(17)1;Gata1^Yeym2^* mice (*n* = 5). ^*^p < 0.05; ^**^p < 0.01.

To further characterize the cells from bone marrow and spleen of the mouse mutants, we performed flow cytometric analysis. Consistent with the histological analysis, our flow cytometric data indicate an increase of cells from CD41^+^ megakaryocyte lineage in bone marrow and spleen of both *Dp(10)1;Dp(16)1; Dp(17)1* and *Dp(10)1;Dp(16)1;Dp(17)1;Gata1^Yeym2^* mice (Figure 7A). These data support the conclusion that both *Dp(10)1;Dp(16)1;Dp(17)1* and *Dp(10)1;Dp(16)1;Dp(17)1;Gata1^Yeym2^* mice developed megakaryocytosis. There is no significant difference in the percentages of CD41^+^ megakaryocyte lineage cells between *Dp(10)1;Dp(16)1;Dp(17)1* and *Dp(10)1;Dp(16)1;Dp(17)1*;*Gata1^Yeym2^* mice (Figure 7A). In addition, Ter119^+^ erythrocyte lineage cells were reduced in the bone marrows of both *Dp(10)1;Dp(16)1;Dp(17)1* mice and *Dp(10)1;Dp(16)1;Dp(17)1*;*Gata1^Yeym2^* mice (Figure 7B). Ter119^+^ erythrocyte lineage cells were increased in the spleens of both *Dp(10)1;Dp(16)1;Dp(17)1* mice and *Dp(10)1;Dp(16)1;Dp(17)1*;*Gata1^Yeym2^* mice, and such increases are likely associated with splenomegaly.

### Triplications of all Hsa21 orthologous regions in mice result in perturbations of hematopoietic stem cells as well as expansion of megakaryocyte-erythroid progenitors and reduction of granulocyte-macrophage progenitors

**Figure 8:**
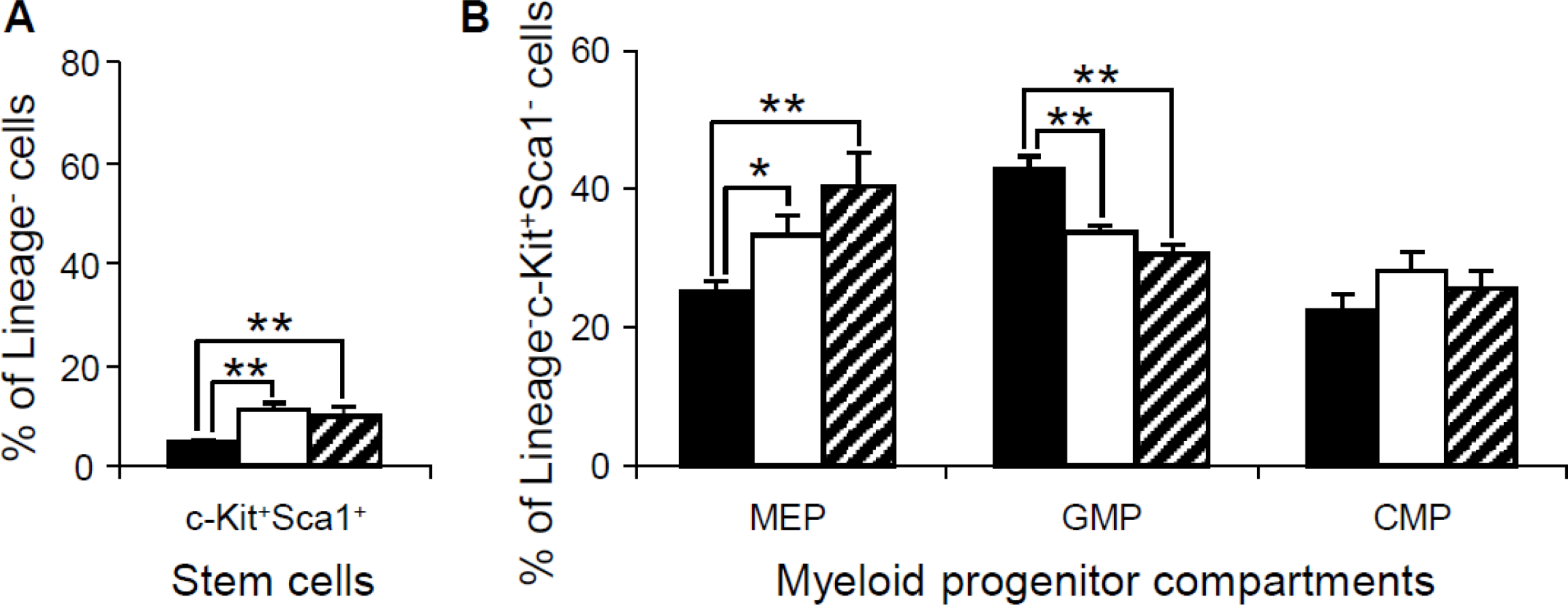
Flow cytometric analysis of the stem cells and myeloid progenitor compartments in bone marrow of wild-type controls, *Dp(10)1;Dp(16)1;Dp(17)1* mice and *Dp(10)1;Dp(16)1;Dp(17)1;Gata1^Yeym2^* mice (**A**) Stem cells (lineage^−^, Sca1^+^, c-Kit^+^) (LSK); (**B**) Myeloid progenitor compartments: MEPs (lineage^−^, Sca1^−^, c-Kit^+^, CD34^−^, FcγR^−^), GMPs (lineage^−^, Sca1^−^, c-Kit^+^, CD34^+^, FcγR^+^) and CMPs (lineage^−^, Sca1^−^, c-Kit^+^, CD34^+^, FcγR^−^). Solid bar, the wild-type controls (*n* = 6); open bar, *Dp(10)1;Dp(16)1;Dp(17)1* mice (*n* = 6) and diagonal-lined bar, *Dp(10)1;Dp(16)1;Dp(17)1;Gata1^Yeym2^* mice (*n* = 5). ^*^p < 0.05; ^**^p < 0.01.

To further determine whether the abnormalities in the peripheral blood, spleen and bone marrow were associated with the changes in hematopoietic stem cells or progenitor populations in bone marrow, flow cytometry was performed to examine the lineage^-^Sca1^+^c-Kit^+^ (LSK) hematopoietic stem cells and myeloid progenitors in the bone marrow of 15-month-old *Dp(10)1;Dp(16)1;Dp(17)1* mice, *Dp(10)1;Dp(16)1;Dp (17)1;Gata1^Yeym2^* mice, and wild-type controls. Our results showed that the LSK stem cells were expanded in both *Dp(10)1;Dp(16)1;Dp(17)1* mice and *Dp(10)1;Dp(16)1; Dp(17)1;Gata1^Yeym2^* mice compared to the wild-type controls (Figure 8A). In myeloid progenitor compartments, the common myeloid progenitors (CMPs) (lineage^−^, Sca1^−^, c-Kit^+^, CD34^+^, FcγR^−^) remained unchanged, while the megakaryocyte-erythrocyte progenitors (MEPs) (lineage^−^, Sca1^−^, c-Kit^+^, CD34^−^, FcγR^−^) and the granulocyte-monocyte progenitors (GMPs) (lineage^−^, Sca1^−^, c-Kit^+^, CD34^+^, FcγR^+^) were increased and decreased, respectively, in both *Dp(10)1;Dp(16)1;Dp(17)1* mice and *Dp(10)1;Dp(16)1;Dp(17)1;Gata1^Yeym2^* mice when compared to the wild-type controls (Figure 8B). There were no significant differences in the LSK stem cells, MEPs, GMPs and CMPs between *Dp(10)1;Dp(16)1;Dp(17)1* mice and *Dp(10)1;Dp(16)1;Dp(17)1;Gata1^Yeym2^* mice (Figure 8).

### Triplication of the Hsa21 orthologous region on Mmu16 results in expansion of MEPs and reduction of GMPs

**Figure 9:**
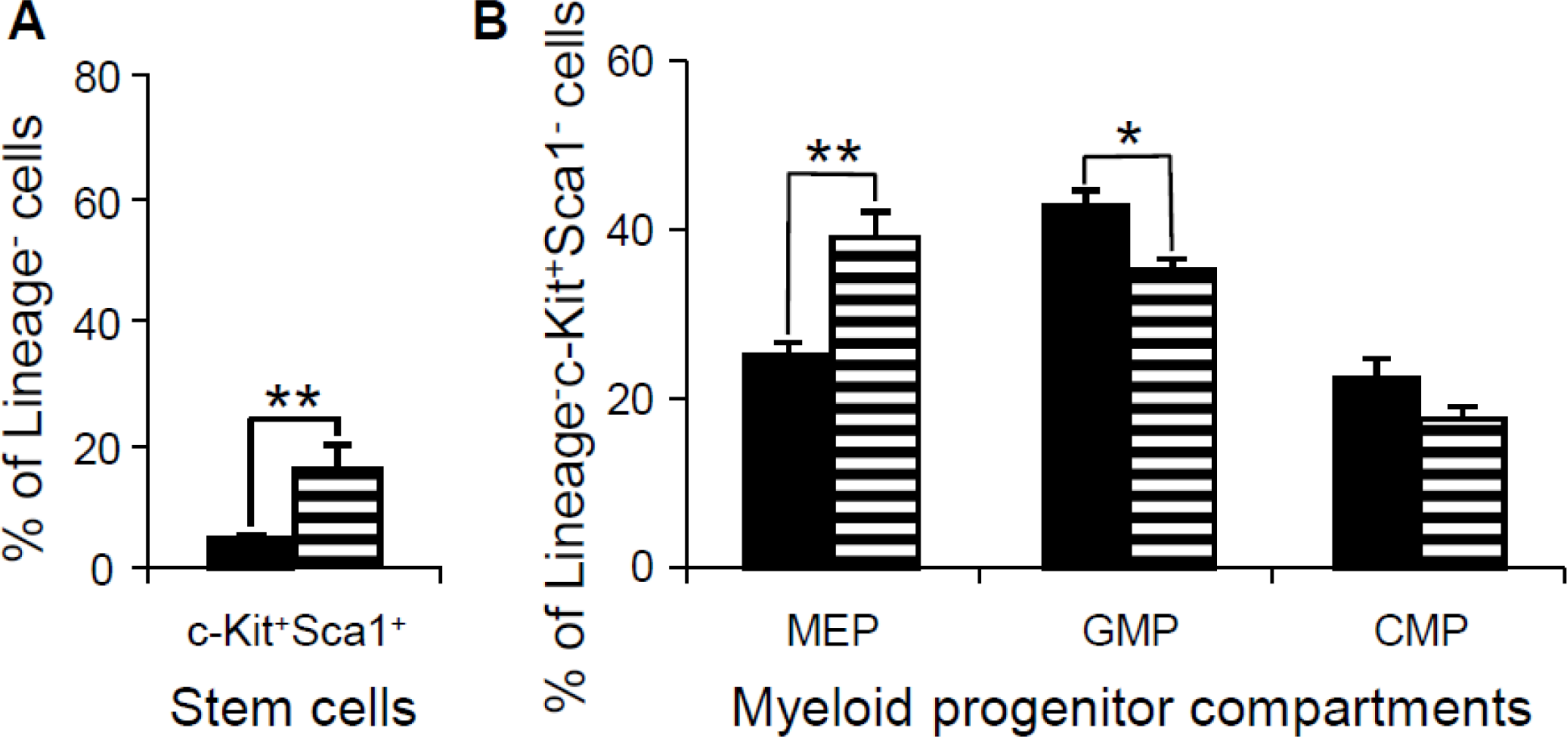
Flow cytometric analysis of the stem cells and myeloid progenitor compartments in bone marrow of wild-type controls and *Dp(16)1* mice (**A**) Stem cells (lineage^−^, Sca1^+^, c-Kit^+^) (LSK); (**B**) Myeloid progenitor compartments: MEPs (lineage^−^, Sca1^−^, c-Kit^+^, CD34^−^, FcγR^−^), GMPs (lineage^−^, Sca1^−^, c-Kit^+^, CD34^+^, FcγR^+^) and CMPs (lineage^−^, Sca1^−^, c-Kit^+^, CD34^+^, FcγR^−^). Solid bar, the wild-type controls (*n* = 6); horizontal-lined bar, *Dp(16)1* mice (*n* = 5). ^*^p < 0.05; ^**^p < 0.01.

In contrast to *Dp(10)1;Dp(16)1;Dp(17)1* mice, Ts65Dn mice have repeatedly shown reduction of MEPs and expansion of GMPs [19, 39]. Ts65Dn and *Dp(16)1* carry 3 copies of the identical set of 100 Hsa21 gene orthologs on Mmu16, so we speculated that *Dp(16)1* mice would likely also show reduction of MEPs and expansion of GMPs. We further speculated that expansion of MEPs and reduction of GMPs in *Dp(10)1;Dp(16)1;Dp(17)1* mice may be caused by *Dp(10)1* and/or *Dp(17)1*. To test these possibilities, we carried out phenotypic analysis of *Dp(16)1* mice. To our surprise, the hematopoietic phenotypes of *Dp(16)1* mice are almost identical to those of *Dp(10)1;Dp(16)1;Dp(17)1* mice, including expansion of MEPs and reduction of GMPs (Figure 9 and Supplementary Figures 1–4). These results suggest that the causative gene(s) responsible for expansion of MEPs and reduction of GMPs are located in the Hsa21 orthologous region on Mmu16. These results also suggest that triplicated genes which differ between *Dp(16)1* and Ts65Dn mice are responsible for diametrically opposite effect on MEPs and GMPs.

## DISCUSSION

**Figure 10:**
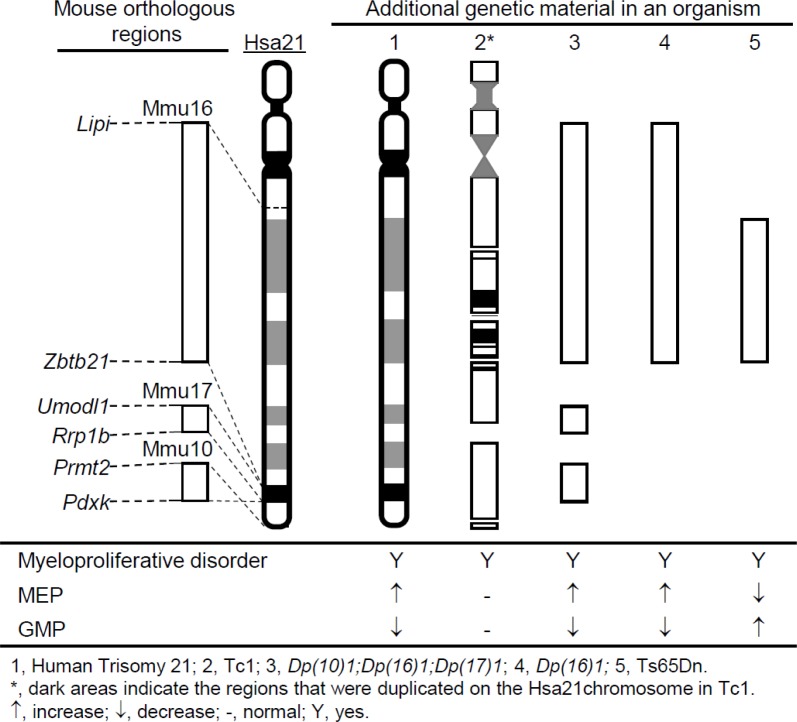
The status of the myeloid progenitors in the major segmental chromosomal alteration mouse models of DS showing myeloproliferative disorder

In this study, we analyzed the hematopoietic phenotype of the *Dp(10)1;Dp(16)1;Dp(17)1* mouse model of DS, which contains duplications spanning the entire Hsa21 orthologous regions on Mmu10, Mmu16, and Mmu17 [15, 28]. These mice exhibited anemia, macrocytosis, thrombocytosis, megakaryocytosis, splenomegaly, and perturbed hematopoietic stem cells and progenitor cell compartments including expanded MEP and reduced GMP populations (Figure 10). The subsequent analysis of *Dp(16)1* mice indicated that these phenotypes are the consequences of the triplication of Hsa21 gene orthologs on Mmu16.

Hematopoietic phenotypes have been characterized in two other major mouse models of DS, Ts65Dn and Tc1 [18, 19]. Ts65Dn mice carry a triplication of a genomic fragment of approximately 13.5 Mb extending from miR-155 to the telomere on Mmu16 and encompassing 100 Hsa21 gene orthologs, which accounts for approximately 57% of the Hsa21 gene orthologs (Figure 10) [22, 40, 41]. Ts65Dn mice also carry a triplicated genomic fragment of approximately 10 Mb extending from the centromere to *1700010I14Rik* on Mmu17 which contains 36 Hsa6 gene orthologs [40, 41]. Ts65Dn mice, *Dp(10)1;Dp(16)1;Dp(17)1* mice and *Dp(16)1* mice share some major features of the hematopoietic phenotype, such as anemia, macrocytosis and myeloproliferative disease characterized by thrombocytosis, megakaryocytic hyperplasia and a distorted hematopoietic stem cell compartment [19]. However, these three models also exhibit important differences in the hematopoietic features. Compared with the wild-type controls, the percentages of MEPs and GMPs in the myeloid progenitor compartments are increased and decreased, respectively, in both *Dp(10)1;Dp(16)1;Dp(17)1* and *Dp(16)1* mice (Figures 8B, 9B). In contrast, the percentages of MEPs and GMPs are decreased and increased, respectively, in Ts65Dn mice (Figure 10) [19]. Because MEPs are the progenitors of megakaryocytes, this specific observation in *Dp(10)1;Dp(16)1;Dp(17)1* and *Dp(16)1* mice may better reflect the abnormal hematopoiesis underlying DS-associated myeloproliferative disorder. Such reasoning is also supported by the observation that the percentages of MEPs and GMPs are increased and decreased, respectively, in the livers of fetuses carrying human trisomy 21 [42–44]. Tc1 mice carry a Hsa21 with three major deletions and other genomic alterations, including duplications and gene mutations, which are likely caused by irradiation of Hsa21 during microcell-mediated chromosome transfer [26, 45]. The random loss of the transferred human chromosome during mouse development resulted in variable levels of mosaicism of the extra chromosome in different tissues [26]. Tc1 mice, *Dp(10)1;Dp(16)1;Dp(17)1* and *Dp(16)1* mice share some important features of the hematopoietic phenotype, such as anemia, macrocytosis, splenomegaly, and an increase in the numbers of megakaryocytes in the spleen [18]. However, unlike *Dp(10)1;Dp(16)1;Dp(17)1* and *Dp(16)1* mice, Tc1 mice did not exhibit significant abnormalities in hematopoietic stem cell and myeloid progenitor compartments, such as MEPs and GMPs [18]. The differences in these phenotypic features could be caused by the Hsa21 genes deleted or mutated on the transchromosome, by transchromosome mosaicism, and/or by species-specific differences between the proteins encoded by human and mouse chromosomes. Therefore, *Dp(10)1;Dp(16)1;Dp(17)1* mice exhibit more important DS-patient-associated hematopoietic abnormalities than other major mouse models of DS carrying an additional copy of Hsa21 orthologous regions, particularly expansion of MEPs and reduction of GMPs. These mice will provide a more desirable system for a better understanding of disease processes and the underlying pathogenic mechanism.

It is a surprise that *Dp(16)1* and Ts65Dn exhibit opposing MEP and GMP phenotypes since they share the same 100 triplicated genes. However, there are additional triplicated genes which differ between the two models: *Dp(16)1* carries a triplication of 15 additional Hsa21 gene orthologs on Mmu16, while Ts65Dn carries a triplication of 36 additional Hsa6 gene orthologs on Mmu17. Therefore, these genetic differences may underlie the opposite phenotypes related to MEPs and GMPs.

MEPs are the myeloid progenitors of megakaryocytes, and therefore the expansion of MEPs observed in human DS and *Dp(10)1;Dp(16)1;Dp(17)1* and *Dp(16)1* mice could be a major logical cellular event leading to myeloproliferative disorder in humans and mouse models (Figure 10). However, Ts65Dn mice also exhibit myeloproliferative disorder (Figure 10) [19] despite exhibiting a reduction of MEPs. One possible reason for this is that differentiation of MEPs to megakaryoblasts may be further accelerated in Ts65Dn, which may then lead to a relative reduction in the number of MEPs. Regardless of the possible reasons, reduction of MEPs detected in Ts65Dn mice deviates from the hematopoietic abnormality observed in human DS. Thus, unraveling why *Dp(16)1* and Ts65Dn are different in MEP- and GMP-related phenotypes may help to improve our understanding of the mechanism underlying myeloproliferative disorder in DS.

Expansion of MEPs and reduction of GMPs were also observed in compound transgenic mice which carry a human *ERG* transgene and a germline *Gata1s* mutation [46]. The *ERG* transgene was driven by regulatory elements from the mouse *vav* gene. There are several potential reasons why this compound mutant may not accurately model the related pathogenic process in human DS. First, the cell- and temporal-specific expression patterns of *vav* and *Erg* may be different, so the expression pattern of the *ERG* transgene may deviate from that of the endogenous *Erg*. Second, the levels of expression between the wild-type allele of *Erg* and the transgenic *ERG* may be also different. A more desirable approach to assess the contribution of an individual Hsa21 gene ortholog to a phenotype is to use a “subtractive strategy”, which involves normalizing the dosage of a Hsa21 gene ortholog by compounding its nonfunctional allele in a segmental chromosomal alteration mouse model of DS. When this strategy was applied to *Erg* in Ts65Dn mice, reducing *Erg* from 3 copies to 2 copies actually led to a significant expansion of MEPs [39], which indicates that the increased *Erg* copy number in Ts65Dn mice is responsible for the reduction of MEPs. This result suggests the *ERG/Gata1s* transgenic mice described above may not be appropriate to model the MEP expansion in human DS.

In normal human hematopoietic cells, both full length GATA1 and GATA1s protein are expressed [47]. Germline and somatic mutations have been detected in exon 2 of the *GATA1* gene, which resulted in expression of only the GATA1s protein without GATA1. The somatic mutations are associated with DS-TMD and AMKL in children with DS [6, 8, 48–51]. The human germline *GATA1s* mutation has been shown to cause anemia in males by affecting erythrogenesis [8]. This mutation-associated effect is also reflected in *Dp(10)1;Dp(16)1;Dp(17)1;Gata1^Yeym2^* mice, since they exhibited more severe anemia than *Dp(10)1;Dp(16)1;Dp(17)1* mice (Figures 3A, 3B). In humans, the germline *GATA1s* mutation affects the morphology of platelets but not their number in the peripheral blood [8]. The impact of the *Gata1s* mutation on the platelets of our mouse models is reminiscent of that seen in humans; there is no significant difference in the number of platelets in the CBC between *Dp(10)1;Dp(16)1;Dp(17)1* and *Dp(10)1;Dp(16)1;Dp(17)1;Gata1^Yeym2^* mice, but the mean platelet volume is expanded in *Dp(10)1;Dp(16)1;Dp(17)1;Gata1^Yeym2^* mice when compared to *Dp(10)1;Dp(16)1;Dp(17)1* mice (Figures 3E, 3F).

In this study, we demonstrated expansion of MEPs and reduction of GMPs in major mouse models of DS with human trisomy 21-associated segmental genomic alterations for the first time, mirroring the corresponding changes of these myeloid progenitors in DS. Thus, further study of these models may lead to identification of the causative gene(s) for expansion of MEPs, which will be the key to a better understanding of the pathogenic mechanism of DS-associated myeloproliferative disorder. In particular, these models may provide further insight into how trisomy 21 leads to expansion of MEPs and how the quantitative alteration of MEPs contributes to the development of myeloproliferative disorder in DS.

## MATERIALS AND METHODS

### Generation of the *Gata1^Yeym2^* mutant mice

The mutant mouse strain was generated using gene-targeting in ES cell to delete exon 2 of the *Gata1* gene which resulted in the expression of a truncated mutant protein, GATA1s. The targeting vector, pTV*Gata1*, was constructed by inserting two homologous regions into a Bluescript KS plasmid that contained a *PGKneobpA (loxP)* cassette in the polylinker (a gift from Dr. Richard Behringer). The first homology region was amplified by primers N1033 (5′-TGCGGCCGCTGGCCCTGATCTCAGCTCAGAATA-3′) and B3614 (5′-TGGATCCTACCGCCCCATTTGTACCAATCCT-3′) to produce a 2598-bp fragment in between intron 2 and intron 5. After cloning the first fragment into pCR-XL-TOPO^®^ using TOPO^®^XL PCR Cloning Kit (Invitrogen), the homology arm was excised out by digesting with *Not*I and *Bam*HI. The second homology region was amplified by primers H4204 (5′-TTAAGCTTCCACCACCGCCTGGCTCTTC-3′) and K7468 (5′-TGGTACCAGTGCTGGGTTTAAAGGTGTGC-3′) to produce a 3286-bp fragment inside the first intron. After cloning the second fragment into pCR-XL-TOPO^®^, the homologous arm was excised out by digesting with *Hind*III and *Kpn*I. The excised fragments were then sequentially inserted between *Not*I and *Bam*HI site as well as between *Hind*III and *Kpn*I sites of the ploylinkers on either side of the *PGKneobpA(loxP)* cassette. The resulting targeting vector, pTV*Gata1*, was linerized with *Kpn*I and electroporated into AB2.2 ES cells. The targeted allele, *Gata1^Yeym1^*, was obtained by G418 selection as described previously [52, 53]. Cre/*loxP*-mediated marker excision [54] was carried out and the final excised allele, *Gata1^Yeym2^*, was identified by Southern blot analysis with *Bam*HI digested DNA. The probe used in the Southern blot hybridization was amplified by primers Probe-U (5′-GGGGGACATTGGGACAGAATAGTT-3′) and Probe-L (5′-ATTCCCCCACGCTCCTATCTCAT-3′), which is mapped to the genomic region downstream of the homologous region in Intron 1 (Figure 2).

### Mice

*Gata1^Yeym2^* mice were first established in a 129SvEvxC57BL/6JF1 strain background and then backcrossed to C57BL/6J mice for five generations. *Dp(10)1,* *Dp(16)1*, and *Dp(17)1* mice were generated using recombinase-mediated genome engineering, which carry the duplications spanning the entire Hsa21 orthologous regions on Mmu10, Mmu16 and Mmu17, respectively [15, 28]. *Dp(10)1*, *Dp(16)1* and *Dp(17)1* mice were backcrossed to C57BL/6J mice for five generations before being used to generate *Dp(10)1;Dp(16)1;Dp(17)1* mice. The latter was crossed to *Gata1^Yeym2^* mice to establish *Dp(10)1;Dp(16)1;Dp(17)1;Gata1^Yeym2^* mice. All mice were maintained at a temperature- and humidity-controlled animal facility. *Gata1* is located on X chromosome. To facilitate appropriate interpretation, we have used only male mice with different genotypes in all the experimental procedures of this study, so only single copy of the *Gata1* or *Gata1s* allele is present in all mice used in the procedures. All the experimental procedures performed were approved by the Institutional Animal Care and Use Committee at Roswell Park Cancer Institute.

### RT-PCR

Total RNA was extracted from the livers of E14.5 embryos using TRIzol Reagent and PureLink RNA Micro kit (Invitrogen Corp., Carlsbad, CA). One μg of the total RNA from each embryo was used to synthesize cDNA by Superscript version III reverse transcriptase (Invitrogen Corp., Carlsbad, CA). To detect the expression of *Gata1* and *Gata1s*, cDNA was analyzed by PCR amplification with the primers GATA1SRTF1 (TCAGAGGCCAAGGCCAGTGAGGACT) and GATA1SRTR1 (TTGCCATAGGCCCAGCTAGCATAAGG). The lengths of the PCR products are 410- and 171-bp, reflecting to the wild-type and the *Gata1^Yeym2^* alleles, respectively (Figure 2C).

### Complete blood counts

Blood samples (∼100 μL) were collected from the retro-orbital sinus using heparinized capillary tubes (Fisher Scientific, Pittsburgh, PA) into Mutlivette 600 K3E vials (SARSTEDT AG & Co., Nümbrecht, Germany) and analyzed on a Hemavet 850 complete blood counter (Drew Scientific, Waterbury, CT).

### Histology

Bones from femur or sternum and spleen were fixed in 10% buffered formalin and were further processed by the Roswell Park Cancer Institute Pathology Core Facility. Bone marrow samples were decalcified prior to processing. Paraffin sections of spleen and bone marrow were stained with hematoxylin and eosin. Slides were photographed on an Olympus BX41 microscope with a DP70 camera and captured with DP Controller software version 1.2.1.108 (Olympus Optical Co., Japan).

### Flow cytometry

Single-cell suspensions from bone marrow or spleen were prepared by passing the tissues through 70 μm cell strainers (BD Pharmingen, San Diego, CA) and red blood cells were lysed in RBC lysis buffer (150 mM NH_4_Cl, 10 mM KHCO_3_, 0.1 mM EDTA). One million cells from each sample were then labeled with the following, anti-CD41-FITC (BD Pharmingen) or anti-TER119-APC-Cy7 (BD Pharmingen) for 30 minutes on ice. Labeled cells were washed in autoMACS Rinsing Solution (Miltenyi Biotec, Aubum, CA) with the addition of 0.5% BSA, fixed with 1% paraformaldehyde in PBS overnight and then analyzed on a LSRII cytometer (BD Biosciences, San Jose, CA). For analysis of Lineage^-^Sca1^+^c-Kit^+^ (LSK) cell and myeloid progenitor cells, ten million bone marrow cells were lineage depleted by using the Lineage Cell Depletion Kit (Miltenyi Biotec) according to the manufacturer's protocol with the addition of the anti-IL-7R-biotin antibody (BD Pharmingen). After magnetic separation of the lineage cells through the MACS MS column, the lineage negative cells were incubated with streptavidin-PE-Cy5.5 (eBioscience, San Diego, CA), anti-Sca1-PE-Cy7 (BD Pharmingen), anti-c-Kit-APC (BD Pharmingen), anti-FcγR-PE (BD Pharmingen) and anti-CD34-FITC (BD Pharmingen) on ice for 30 minutes and analyzed on a LSRII cytometer after washing and fixation as described above.

### Statistical analysis

Statistical analysis was performed using Student *t*-test and the non-parametric Mann-Whitney *U*-test. The level of statistical significance was set at *p* = 0.05. Data are presented as Mean ± SEM.

## SUPPLEMENTARY MATERIALS FIGURES


