# Supplementary material for: Triplications of human chromosome 21 orthologous regions in mice result in expansion of megakaryocyte-erythroid progenitors and reduction of granulocyte-macrophage progenitors
Source: Oncotarget. 2017 Dec 19;9(4):4773–86. doi: 10.18632/oncotarget.23463 (PMC5797011; doi:10.18632/oncotarget.23463)
Supplement: Supplementary file 1 [file oncotarget-09-4773-s001.pdf]

Triplications of human chromosome 21 orthologous regions in mice result in expansion of megakaryocyte-erythroid progenitors and reduction of granulocyte-macrophage progenitors

SUPPLEMENTARY MATERIALS

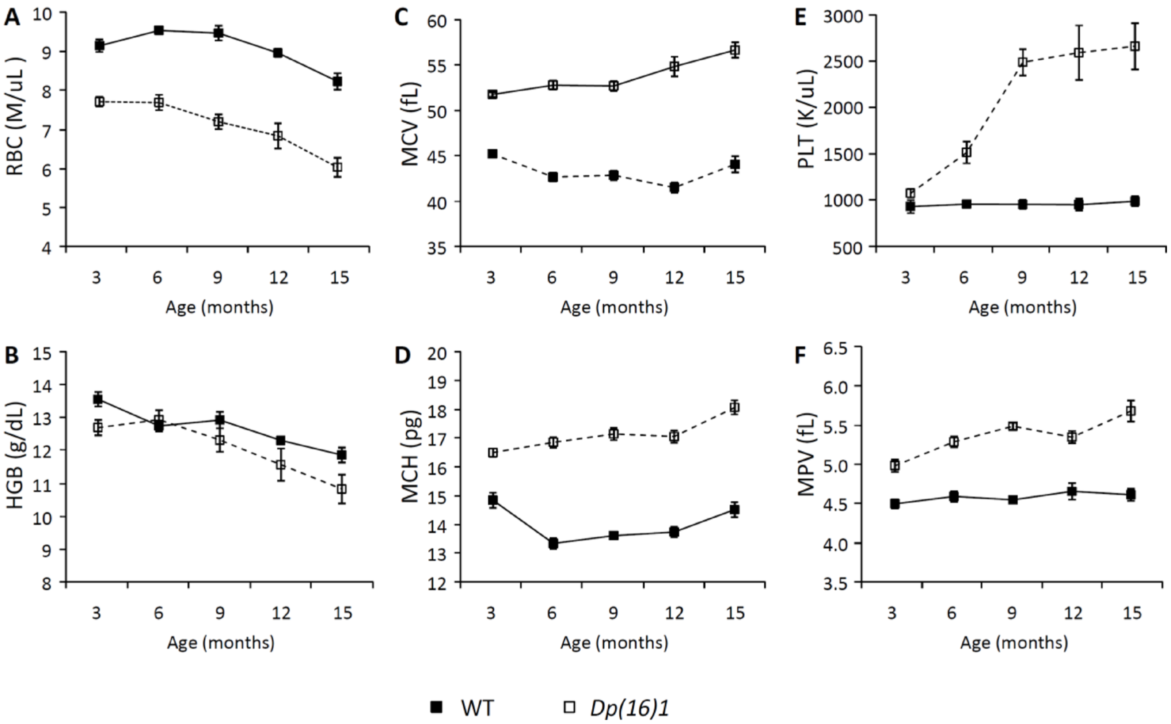

Supplementary Figure 1: Complete blood counts (CBC) of *Dp(16)1* mice and wild-type controls. (**A**) Red blood cells (RBC) (M/ µL; millions per microliter); (**B**) Hemoglobin (HGB) (g/dL, grams per deciliter); (**C**) Mean corpuscular volume (MCV) (fL, fentoliters); (**D**) Mean corpuscular hemoglobin (MCH) (pg, picograms); (**E**) Platelets (PLT) (K/µL; thousands per microliter); (**F**) Mean platelet volume (MPV). Open square, Dp(16)1 mice (*n* = 15, 12, 14, 14, 13 at 3, 6, 9, 12, 15 months of age, respectively); closed square, wild-type controls (*n* = 14, 14, 9, 15, 21 at 3, 6, 9, 12, 15 months of age, respectively).

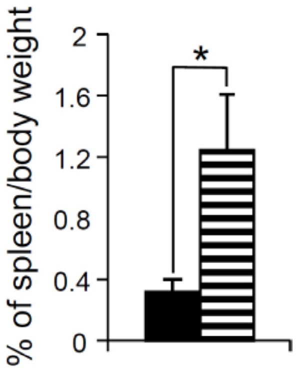

Supplementary Figure 2: Relative weights of the spleens from *Dp(16)1* mice and wild-type controls. The percentage of spleen weight over body weight in wild-type controls ($n = 6$, solid bar) and *Dp(16)1* mice ($n = 6$, horizontal-lined bar). $^{*}P < 0.05$.

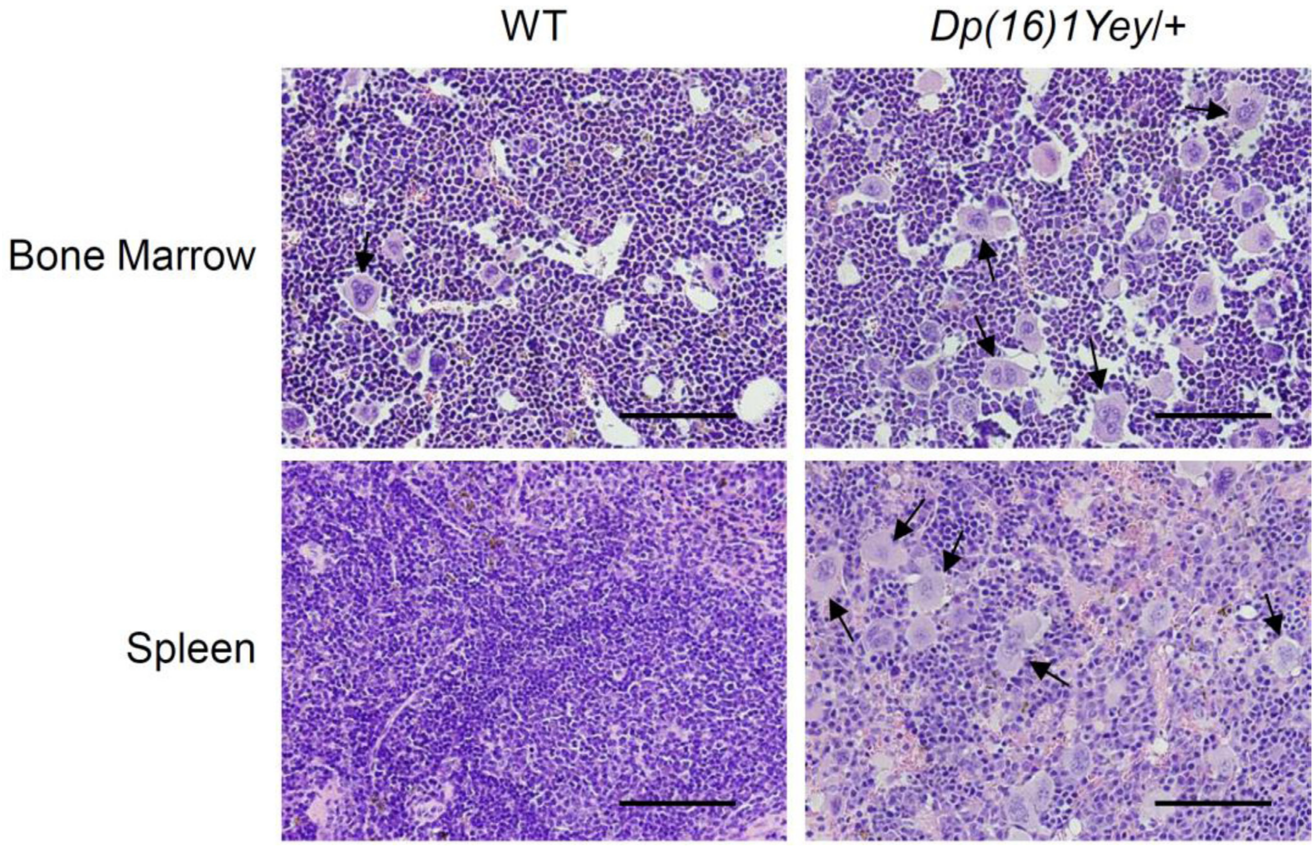

Supplementary Figure 3: Histological sections of the bone marrow and spleens from *Dp(16)1* mice. Hematoxylin and eosin staining of bone marrow and spleen sections in wild type controls and *Dp(16)1* mice. Scale bar, 10 µm. Arrows point to the megakaryocytes in the sections.

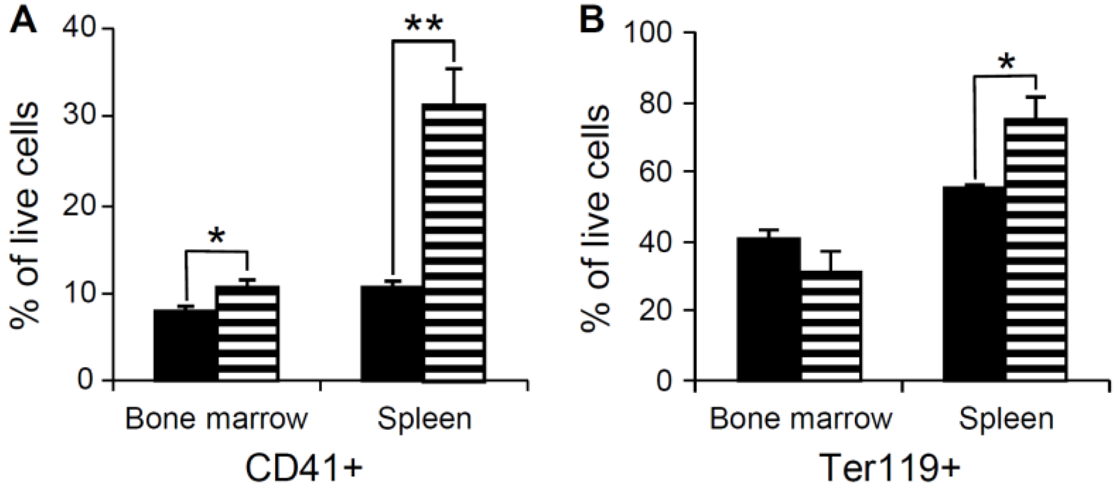

Supplementary Figure 4: Flow cytometry analysis of bone marrow and spleen cells in wild type controls and *Dp(16)1* mice. (**A**) CD41+ megakaryocyte lineage cells; (**B**) Ter119+ erythrocyte lineage cells. Solid bar, wild type controls (n = 6 & 4 in panel A & B. respectively) and horizontal-lined bar, *Dp(16)1* mice (n = 5 & 3 in panel A & B, respectively). $^{*}P < 0.05$; $^{**}P < 0.01$.
